# The potential of robot eyes as predictive cues in HRI—an eye-tracking study

**DOI:** 10.3389/frobt.2023.1178433

**Published:** 2023-07-28

**Authors:** Linda Onnasch, Paul Schweidler, Helena Schmidt

**Affiliations:** ^1^ Technische Universität Berlin, Berlin, Germany; ^2^ HFC Human-Factors-Consult GmbH, Berlin, Germany; ^3^ Land in Sicht—PROWO gGmbH, Eberswalde, Germany

**Keywords:** human-robot interaction (HRI), attentional processes, joint attention, anthropomorphism, robot design

## Abstract

Robots currently provide only a limited amount of information about their future movements to human collaborators. In human interaction, communication through gaze can be helpful by intuitively directing attention to specific targets. Whether and how this mechanism could benefit the interaction with robots and how a design of predictive robot eyes in general should look like is not well understood. In a between-subjects design, four different types of eyes were therefore compared with regard to their attention directing potential: a pair of arrows, human eyes, and two anthropomorphic robot eye designs. For this purpose, 39 subjects performed a novel, screen-based gaze cueing task in the laboratory. Participants’ attention was measured using manual responses and eye-tracking. Information on the perception of the tested cues was provided through additional subjective measures. All eye models were overall easy to read and were able to direct participants’ attention. The anthropomorphic robot eyes were most efficient at shifting participants’ attention which was revealed by faster manual and saccadic reaction times. In addition, a robot equipped with anthropomorphic eyes was perceived as being more competent. Abstract anthropomorphic robot eyes therefore seem to trigger a reflexive reallocation of attention. This points to a social and automatic processing of such artificial stimuli.

## 1 Introduction

Industrial collaborative robots, or cobots for short, interact in direct temporal and physical proximity with a human partner (Restrepo et al., 2017). The accompanying elimination of safety barriers creates new requirements for coordination and action prediction between humans and robots. To date, however, cobots only provide limited information about future motion sequences, making it a hard task for humans to coordinate their behavior around the cobot–especially compared to how easy it is for humans to coordinate their interpersonal behavior. Explicit predictive cues would seem to be a good idea to make the robot’s movements easier to understand. Yet, compared to social robots or other service robots, the design space offered by industrial cobots is quite a narrow one, as it is bounded rather by the specifications of the industrial task, performance metrics and a functional design, than by the affordances of a fluent human-robot interaction (HRI). If we want to implement predictive cues, we argue they have to meet at least three requirements. First, their implementation must not conflict with the robot’s performance: e.g., Faria et al. (2021) proposed a solution to make robotic movements more legible to the operator, but this was at the expense of extra costs in motion planning. Second, the predictive cues need to fit into the functionalist design scope. Thus, a simple, straight forward approach would come to mind, like the use of arrows on a screen to indicate motion intention of a mobile robot (Shrestha et al., 2016), or projected arrows on the ground (Hetherington et al., 2021). Another design option that has been explored in this regard are moving lightbands to indicate motion intents of a mobile factory robot (Bacula et al., 2020). Third, as industrial human-robot coordination is not the main part of task fulfillment but rather a means to an end, the predictive cues should trigger resource-efficient mechanisms that do not require additional cognitive resources (Neider et al., 2010). This means that humans’ attention shifts required to predict the robotic motion should happen as effortlessly as possible, i.e., automatically (Onuki et al., 2013; Khoramshahi et al., 2016). Arrows as indicators for robot movements might not fulfill this requirement as the interpretation of these cues needs an active consideration and therefore additional cognitive resources.

To find a solution integrating all these requirements, we think that functional anthropomorphic features, i.e., abstract forms of anthropomorphism that only aim to mimic certain functional aspects of human-likeness, are a promising suspect (Onnasch and Roesler, 2021). One such feature is the attention directing function of eyes and gaze. In human interaction, eye gaze is a key mechanism to engage in joint attention, which describes an automatic reallocation of one’s attention to an object that another individual is attending to (Shepherd, 2010). This, in turn, enables us to understand, predict and adapt to the situation. The automaticity in joint attention is very resource efficient as it does not require an active interpretation of the directional gaze information and thereby does not interfere with other cognitively demanding activities. Accordingly, the implementation of abstract anthropomorphic eyes into robot design might be a resource efficient option to make robot movements more predictable. However, there is evidence that only social stimuli evoke joint attention in contrast to non-social stimuli like arrows (Ricciardelli et al., 2002; Friesen et al., 2004; Ristic and Kingstone, 2005). Whether abstract anthropomorphic eyes like robot eyes, trigger joint attention has therefore been the subject of several studies, which point to a great potential (Admoni and Scassellati, 2017). People have no problems reliably following a robot’s gaze (e.g., Wiese et al., 2018; Onnasch et al., 2022), and use it to predict target positions before these are verbalized (Boucher et al., 2012). Even people’s decision-making can be influenced by a robot’s gaze. Mutlu et al. (2009) and Staudte and Crocker (2008) could show that although participants were told to only consider verbal cues, their attention allocation and object selection was biased by a robot briefly gazing at a certain object. Furthermore, human-like gaze trajectories implemented on a robot’s display have the potential to make object handovers of a robotic arm more pleasant and fluid as well as time-efficient (Moon et al., 2014). Similarly, supportive gaze has been shown to improve performance in an interactive map-drawing task and to reduce the cognitive resources required by the human interaction partner (Skantze et al., 2013). However, also detrimental effects of robot eyes are possible when the eyes and according gaze behavior are purely decorative features and do not correspond to the robot’s motion (Onnasch and Hildebrandt, 2021). In such cases, implementing abstract anthropomorphic eyes into robot design has the potential to distract people from their main task and to make interaction more difficult instead of supporting it.

**FIGURE 1:**
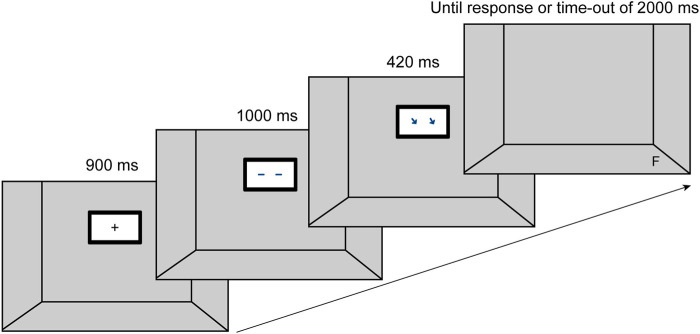
Set-up and sequence of events on a given valid trial. (Figure adapted from Onnasch et al., 2022).

Besides the growing body of evidence showing the effectiveness (or at least attention-grabbing effect) of robotic gaze, it is still unclear to what extent it is really automatic, i.e., to what extent abstract anthropomorphic gaze triggers reflexive attentional shifts. For example, Admoni et al. (2011) could not find a reflexive cueing for robotic stimuli. The study used the Posner paradigm (Posner, 1980), an experimental set-up for spatial cueing. Participants had to look at a fixation cross, which was then replaced by a spatial cue indicating the position of a subsequently following target stimulus to which participants had to react by an according key press as fast as possible (see also Figure 1). Results showed that participants could infer directional information from the robot’s gaze, but they did not reflexively reallocate their attention to the cued position (Admoni et al., 2011). Other studies suggest an automatic attention cueing of robot gaze (e.g., Boucher et al., 2012). Specifically, Chaminade and Okka (2013) found that both human faces and those of a humanoid robot (Nao) led to automatic attentional shifts, Wiese et al. (2018) showed that eye movements of a social robot (Meka) triggered automatic attention-directing effects, and Pérez-Osorio et al. (2018) successfully replicated the gaze cueing effect using a humanoid robot (iCub). However, it is noteworthy that none of these studies explored an isolated use of eye movements, but a more ecologically typical integration of eye movements, head movements and/or pointing gestures. Some of them (Admoni et al., 2011; Chamindae and Okka, 2013) did not seem to use robots with moving or animated eye parts at all. The specific variance-explaining proportions of gaze thus cannot be determined. Mutlu et al. (2009) investigated the communication of behavioral intentions through robotic eyes without any head movements and found a positive effect of anthropomorphic eyes, but did not include a non-anthropomorphic control condition. Accordingly, it remains unclear whether abstract anthropomorphic robot eyes actually triggered automatic attentional shifts or whether positive effects were only due to the additional information compared to an interaction without any cues.

In summary, empirical evidence seems to favor the assumption of a beneficial effect of abstract anthropomorphic gaze cues. However, given the methodological characteristics of the existing research it remains unclear whether, in line with the cooperative eye hypothesis (Tomasello et al., 2007), eye movements of a robot are sufficient directional cues without head movements or point gestures. Further systematic research comparing anthropomorphic eye stimuli with non-anthropomorphic cue stimuli is therefore needed. In addition, there is a lack of studies specifically for the industrial application area and the associated special requirements mentioned above (functionalist design, straight forward implementation).

According to these requirements, we investigated in a previous study directional stimuli differing in their degree of anthropomorphism to facilitate attentional shifts for the potential use as robot eyes on an industrial robot (Onnasch et al., 2022). The online study used a modified version of the spatial cueing paradigm (Posner, 1980), using either arrows, abstract anthropomorphic eyes or photographed human eyes as directional stimuli. Attentional shifts were measured indirectly as the time from the target onset (in that case the presentation of a single letter) until the according key press. Results supported the assumption that abstract anthropomorphic eyes have the potential to facilitate HRI, as they led to the fastest responses which is indicative for reflexive gaze cueing. Surprisingly and in contrast to hypotheses, the human eyes did not evoke reflexive attentional shifts as evidenced by longer response times. We suspected that the abstract anthropomorphic eyes elicited the desired effects because they were sufficiently human-like and at the same time much easier to perceive than human eyes, with the latter being due to the abstract anthropomorphic eyes’ design featuring strong contrasts and clean lines. This is an interesting finding and may prove helpful for designing better HRI. However, to see whether this is in fact a solid basis for further conclusions and actions, those unexpected findings with regard to the superiority of anthropomorphic, non-human eyes, even in comparison to human eyes, call for a validation. Especially, because the implementation as an online-study comes with a lack of control in terms of standardized situational circumstances and hardware (light conditions, distraction, screen resolution, …). Moreover, the measurement of attention was only realized via covert measures in terms of reaction times. Thus, to further strengthen results and the interpretation that abstract anthropomorphic eyes induce reflexive gaze cueing, the aim of the current study was therefore to validate findings of the previous online study (Onnasch et al., 2022) in a highly controlled laboratory environment and to further deepen insights by introducing direct attentional measures via eye-tracking. We investigated how the design of highly abstract anthropomorphic eyes for a potential use on a collaborative robot should look like in order to reflexively trigger attention reallocation to improve the prediction of robot motion.

## 2 Materials and methods

The experiment was performed with ethical committee approval by the Institute of Psychology, Humboldt-Universität zu Berlin, and in accordance with the Declaration of Helsinki. Informed consent was obtained from each participant. We preregistered the study at the Open Science Framework (osf.io/wue6d).

### 2.1 Participants

A sample size of N = 80 was defined based on an *a priori* power analysis using GPower (Faul et al., 2007; Faul et al., 2009). Due to COVID-19 induced restrictions we had to halve the sample size and recruited 40 participants via the local online recruiting system of the Institute of Psychology, Humboldt-Universität zu Berlin. Participants either received course credit or a €10 compensation at the end of the experiment. One participant had to be excluded because of technical issues. We therefore conducted data analysis with a sample of N = 39 participants with German as native language or equal language abilities (*M* = 32.26 years, *SD* = 10.78 years, 27 females).

### 2.2 Apparatus and task

The experiment was conducted on a 27″ HD Dell Monitor (1,920 × 1,080 px) which was positioned at a distance of 67 cm to a chin rest. The latter was used to minimize artefacts of head movements for eye-tracking data. The setup was a modified version of a traditional spatial cueing paradigm (Posner, 1980; Figure 1) and corresponded to the setup of the previous online study (compare Onnasch et al., 2022). Each trial began with the presentation of a fixation cross in the center of a depicted display on the computer screen (see Figure 1). After 900 ms, a display appeared with a “gaze” facing to the front. 1,000 ms later the gaze averted to a position where the target appeared after a stimulus onset asynchrony (SOA) of 420 ms,. The target disappeared upon participants’ reaction or a time-out of 2000 ms (description taken from Onnasch et al., 2022).

Figure 1 All central cue stimuli as well as the fixation cross were displayed at the subjects’ eye level on the screen. The target stimuli appeared in a 3D-like image of a room. It seems noteworthy at this point that we intentionally designed a screen-based experiment instead of one using a real human-robot interaction. This has been done not only to exclude any confounding effects that the HRI might induce, but also to avoid parallax effects by making the angle between robot eyes and target positions, i.e., the stimulus geometry, absolutely invariant. Nevertheless, to increase ecological validity, we modeled the three-dimensional space with target positions in reference to a physical setup of a shared workspace with an industrial robot (Sawyer by Rethink Robotics). We measured the distances between actual target positions, i.e., positions that the robot could reach with its gripper, the robot’s display, and the human co-worker. These distances were then scaled down and transferred as parameters into our model, that used HTML, JavaScript and raster graphics to render the virtual set-up. Eight different positions were determined for the target stimuli to appear in the experiment. Six target positions were located below the display on what appeared to be a floor (three positions in a front row, three positions in a back row), two target positions were on the side walls, one left and one right, each in a centered position. Implementing eight different target positions represents a significant change from the experimental gaze cueing setup which is conventionally distributed between two positions or a maximum of four positions (e.g., Admoni et al., 2011). This change was deemed necessary to approximate a real industrial HRI situation, thus further increasing ecological validity. The size of the frame in which the fixation cross and cue stimuli were presented centrally covered 7.91° × 4.81° in angle of view (AOV), which corresponds in its relative dimensions to the display of a Sawyer robot. The size of the display of the stimuli in angular degrees was determined approximately oriented to the mean value of previous studies. The cueing stimuli were either images of human eyes, arrows, or two different versions of abstract anthropomorphic eyes (pixel, cross). Following classical gaze cueing tasks, two black sans-serif letters F and T were presented as target stimuli (e.g., Friesen and Kingstone, 1998). These corresponded to 0.50° AOV in their presented size and were presented at a distance of 13.40°–22.75° AOV from the center of the fixation cross, depending on their position in space. The small size of the target stimuli in combination with the high degree of similarity in the typeface of the two letters was to ensure that no discrimination of the target stimuli was possible in the peripheral field of view. It should be necessary to shift the foveal field of view for task performance in order to trigger eye movements of the subjects. Table 1 summarizes the information on the AOV of the respective elements in the experimental set up.

**TABLE 1:** Summary of AOVs of the different elements used in the experimental set-up.

Element	Size in angle of view (AOV)
Frame representing the display	7.91° × 4.81°
Fixation cross	2.60° × 2.60° AOV
Photograph of the human eyes	5.80° × 1.73°
Arrows	1.58° × 1.50° each
Abstract eyes	2.61° × 2.61° each
Letters	0.50°
Distance between letter and fixation cross	between 13.40° and 22.75°

For recording participants’ manual responses to the cue stimuli, the Microsoft Xbox Wireless 1708 controller was used. For the recording of oculomotor movements, the screen-based remote eye tracker model RED500 from iMotion (Senso-Motoric Instruments GmbH, SMI) with a sampling rate of 500 Hz was used. The spatial accuracy of the device amounts to 0.40° for binocular recording, which was also chosen in this study.

### 2.3 Design

**FIGURE 2:**
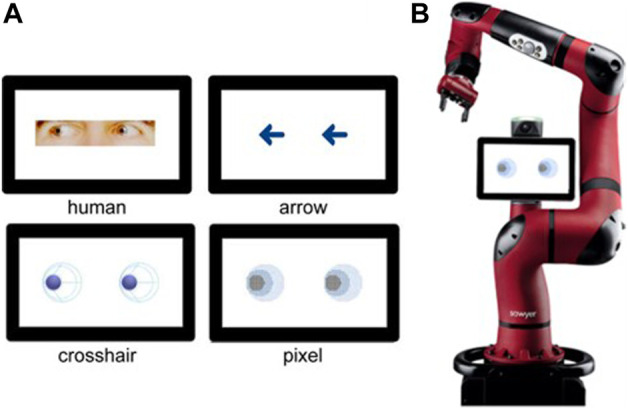
**(A)** The four stimulus types, labelled respectively. The top row includes the human (left) and arrow (right) stimuli. The abstract anthropomorphic robot eyes are presented in the second row. **(B)** Image of the collaborative robot Sawyer used in the questionnaire. In this case, presented featuring the pixel eye design. (Figure adapted from Onnasch et al., 2022).

Two variables were systematically varied in the experiment. First, the cues were varied between-subject, representing either human eyes, abstract anthropomorphic eyes, or arrow stimuli. For the previous online study, the anthropomorphic eyes were designed striving for a maximum level of abstraction while retaining the essential features of the human eye (e.g., visible pupil-sclera size ratio). This resulted in two different anthropomorphic eye designs, that were both exploratively compared in the online study and therefore also implemented in the current laboratory experiment (cross and pixel design, Figure 2). Second, the trial congruency was manipulated as a within-subject factor. From a total of 304 trials, the target stimuli appeared at cued locations in 80% of the trials (240 trials congruent), while in the remaining 20% of trials the target appeared at uncued locations (64 trials incongruent). The distribution of congruent and incongruent trials was defined with a random number generator and was the same in all four conditions. Overall, this resulted in a 4 (stimulus type) × 2 (trial congruency) mixed design. In the previous online study, a third factor was implemented which investigated the impact of paired vs. single stimulus representations (Onnasch et al., 2022). However, as this variation did not have an impact on reaction times, we decided to discard this factor for the follow up study.

### 2.4 Dependent measures

#### 2.4.1 Reaction time

We assessed the reaction times as a covert measure of attention and to evaluate the potential for reflexive cueing of the different stimuli. Reaction times were measured from the target onset to a key press (F or T) on the controller. We only included trials with correct answers (e.g., target F, key press F) as incorrect answers could have biased the results.

#### 2.4.2 Gaze-cueing effect

We calculated the gaze cueing effect (GCE) by subtracting mean reaction times of congruent trials from the mean reaction times of incongruent trials.

#### 2.4.3 Saccadic latency

As an overt attentional measure, saccadic latency was measured. This describes the time elapsing between the appearance of the target letter and the initiation of the orienting saccade away from the cue stimulus. It serves as an indicator of attention directing properties of the cue stimulus and describes how long a disengagement of attention from the cue stimulus took (e.g., Admoni and Scassellati, 2017). Fixations were detected using a dispersion based algorithm with 0.5° and 120 ms as spatial and temporal thresholds. Saccade initiation was defined as the first sample captured outside the fixation area (Nyström and Holmqvist, 2010).

#### 2.4.4 Social attributes

On an explorative basis, we were further interested in how a robot having incorporated the stimulus designs would be perceived. A positive perception of the overall robot design is a crucial precondition for an implementation of such designs in terms of user acceptance. Accordingly, we presented the different stimulus designs as part of an image of an industrial collaborative robot (Sawyer, Rethink Robotics, Figure 2) and asked participants to fill in the Robotic Social Attributes Scale (RoSAS; Carpinella et al., 2017). The RoSAS consists of a total of 18 adjectives and three subscales: warmth, competence and discomfort. Participants have to indicate how closely each adjective is associated with the robot image on a 7-point Likert scale from 1 (definitely not associated) to 7 (definitely associated).

### 2.5 Procedure

Participants were randomly assigned to one of the four between-subject conditions. Upon arrival at the lab, participants received detailed information about the study and data handling. After giving their informed consent, they received instructions for the experiment and started with two training sessions that familiarized them with the task. The first training comprised 12 trials during which a letter (T or F) appeared centrally on the screen. Participants were instructed to place their index fingers on the directional pads of the controller (left shoulder key for F, right shoulder key for T) and to react upon seeing the letters, using the respective keys. The letter changed its color from white to green upon correct response and from white to red, indicating an incorrect reaction.The aim of this training was to get participants used to the key presses without having to shift their gaze to the controller. During the 40 trials of the second training, participants practiced the experimental task. They were told they would look into a room in which a display was hanging at the back wall (see Figure 1). The appearance of a fixation cross started a trial. After each trial an inter-trial interval of 200 ms elapsed before the next trial began. After completing the second training, the main test procedure started, consisting of 304 trials. The training did not include incongruent trials and participants were not told that there would be incongruent trails during the experiment. The time course followed in each trial of the second training and the test procedure is shown in Figure 1. Upon successful completion of the actual experiment, in a last step, participants were asked to fill in remaining questionnaires (sociodemographics & RoSAS). Only for the RoSAS, we presented a contextualized version of the stimulus design as part of the industrial robot Sawyer (Figure 2). The main test (spatial cueing paradigm) was done without depicting a robot but only a screen featuring the stimulus (Figure 1). The entire procedure took approximately 45 min.

## 3 Results

The descriptive data for all three dependent variables are reported for each stimulus condition in Table 2.

**TABLE 2:** Means (and SD) in ms for Reaction Time, Saccadic Latency and Gaze Cueing Effect.

	Reaction time				N
	Cued		Uncued		
Stimulus type	M	SD	M	SD	
Arrow	693.57	51,70	823,67	88,24	9
Pixel	683,28	64,83	802,14	66,81	10
Cross	641,82	66,81	781,23	80,00	9
Human	724,90	76,51	900,05	92,37	11

	Saccadic Latency				N
	Cued		Uncued		
		M	SD	M		SD
Arrow	287,38	18,44	282,45	21,56	9
Pixel	267,63	21,53	297,29	21,39	10
Cross	273,37	11,17	283,07	19,46	9
Human	293,44	17,99	293,71	18,05	11

	Gaze Cueing Effect				N
		M	SD			
Arrow	133,95	45,55			9
Pixel	124,86	45,55			10
Cross	143,83	46,84			9
Human	181,54	61,14			11

### 3.1 Reaction time

**FIGURE 3:**
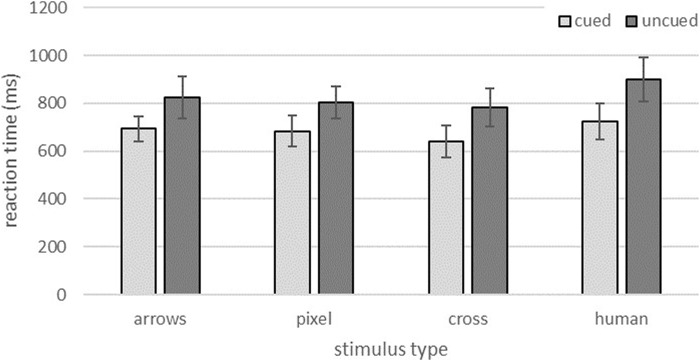
Reaction times for cued and uncued trials for the different stimulus type conditions. Error bars represent standard deviations.

Results are depicted in Figure 3. Reaction times were longer in incongruent trials (*M* = 829.89 ms; *SD* = 97.95 ms) compared to congruent trials (*M* = 687.82 ms; *SD* = 70.36 ms). This was supported by a main effect of trial congruency, *F* (1,70) = 61.91, *p* < 0.001, *η*_*p*_^*2*^ = 0.469.

The data also revealed a significant main effect of stimulus type, *F* (3,70) = 11.78, *p* = 0.001, *η*_*p*_^*2*^ = 0.200. In congruent as well as incongruent trials, the human eyes led on average to the longest reaction times (*M* = 812.47 ms; *SD* = 122.00 ms). The anthropomorphic cross condition elicited the fastest reactions (*M* = 711.52 ms; *SD* = 101.27 ms). No interaction effect was found (*F* < 1).

Bonferroni corrected *post hoc* comparisons showed that only the anthropomorphic cross design (mean difference −100.95 ms, *p* = 0.001) and the pixel design differed significantly from the human eye stimuli (mean difference −53.85 ms, *p* = 0.033) whereas no significant difference emerged between human eyes and arrow stimuli.

### 3.2 Gaze-cueing effect

The mean values for the GCE differed gradually, descriptively decreasing from human stimuli (*M* = 181.54, *SD* = 61.14) over anthropomorphic cross design (*M* = 143.83, *SD* = 46.84) and arrows (*M* = 133.95, *SD* = 45.55) to the anthropomorphic pixel condition (*M* = 124.86, *SD* = 45.55). The univariate ANOVA however did not support this descriptive pattern as no significant main effect of stimulus type was found for GCE, *F* (3,35) = 2.16, *p* = 0.110.

### 3.3 Saccadic latency

**FIGURE 4:**
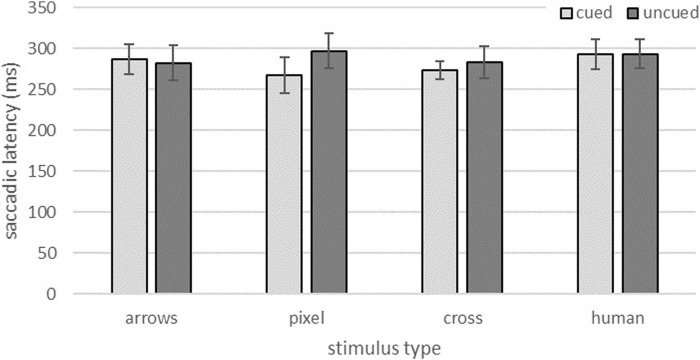
Saccadic latencies for congruent and incongruent trials for the different stimulus type conditions. Error bars represent standard errors.

Similar to the manual reaction times via key press, human eyes appeared to produce the longest (visual) reaction times in both congruency conditions (Figure 4; *M*_*congruent*_ = 293.44 ms; *SD*_*congruent*_ = 17.99 ms; *M*_*incongruent*_ = 293.71 ms; *SD*_*incongruent*_ = 18.05 ms). The descriptive data of the congruent trials also indicated the second longest times for the arrow eyes (*M* = 287.38 ms; *SD* = 18.44 ms) and, at some distance, the anthropomorphic stimuli both followed at about the same level with the lowest values (*M*_*cross*_ = 273.37 ms; *SD*_*cross*_ = 11.17 ms; *M*_*pixel*_ = 267.63 ms; *SD*_*pixel*_ = 21.53 ms). For incongruent trials, the difference between the arrow condition (*M* = 282.45 ms; *SD* = 21.56 ms) and the abstract anthropomorphic eyes (*M* = 293.71 ms; *SD* = 18.05 ms) appeared less evident. Overall, saccadic latency plausibly appeared to be independent of trial congruency.

The two-factorial ANOVA did not show a significant impact of trial congruency on saccadic latencies, *F* (1,70) = 0.94, *p* = 0.337, but a significant effect of stimulus type, *F* (3,70) = 4.40, *p* = 0.007, *η*_*p*_^*2*^ = 0.159. Bonferroni corrected *post hoc* pairwise comparisons detailed this effect and revealed significant differences only between the human stimuli and the anthropomorphic pixel design (*p* = 0.006). All other comparisons did not reach significance.

### 3.4 Social attributes

Results of the RoSAS are displayed in Table 3. On the *warmth* dimension, participants rated the two anthropomorphic stimulus designs highest while arrows received the overall lowest ratings. The ANOVA, however, did not reveal significant differences between the conditions, *F* (3,35) = 2.33, *p* = 0.091.

**TABLE 3:** Cronbach’s alpha, mean ratings (and SD) for the RoSAS.

	Warmth		Competence		Discomfort		
Cronbach’s alpha	0.85		0.83		0.90		
Stimulus Type	M	SD	M	SD	M	SD	N
Arrow	2.00	0.77	4.77	1.14	2.11	0.86	9
Pixel	3.23	1.21	4.15	1.08	2.85	1.52	10
Cross	3.04	0.74	5.61	0.51	2.00	1.27	9
Human	2.79	1.35	4.88	0.95	2.33	1.08	11

The perceived *competence* subscale showed substantial differences for the stimulus designs, *F* (3,35) = 3.72, *p* = 0.020, *η*_*p*_^*2*^ = 0.242. This was due to the high competence ratings of the cross design. Post hoc tests with Bonferroni correction further showed a significant difference of this design compared to the pixel design that was perceived least competent (*p* = 0.010).

With regard to the perceived *discomfort* of the overall robot’s design, the different stimulus types did not significantly change participants’ perception, *F* (3,35) = 0.93, *p* = 0.436.

## 4 Discussion

This study aimed to validate findings of a previous online study on the effectiveness of different directional stimuli regarding reflexive attention allocation (Onnasch et al., 2022) in a highly controlled laboratory environment and to further deepen insights by introducing direct attentional measures via eye-tracking. Both studies investigated how directional stimuli should be designed for a potential use on a collaborative industrial robot to enable human interaction partners to predict the robot’s movements in a cognitively efficient way.

As expected, and in line with the previous online study, a congruency effect could be demonstrated for all four stimulus types. Subjects reacted faster to targets that were correctly indicated by the gaze direction of the stimuli (cued trials) than to those indicated in the opposite direction (uncued trials). This means that all stimulus types were essentially able to support the subjects’ attentional orientation. Such a congruency effect has been demonstrated several times before for different directional cueing stimuli (e.g., Admoni et al., 2011; Chaminade and Okka, 2013; Wiese et al., 2018).

However, a closer look at the reaction times revealed surprising differences in how efficiently the guidance of the subjects’ attention could be supported. Whereas no differences emerged for the GCE, the two abstract anthropomorphic eye designs each resulted in the shortest reaction times in cued trials. The current findings therefore support results from the previous online study, which also revealed the fastest reaction times for the abstract anthropomorphic eyes. In the current study, these findings were further underlined by the subjects’ eye movements. For both anthropomorphic stimulus designs saccadic latencies were descriptively shorter compared to the arrows and the human eyes. A significant difference to the human eye design emerged however, only for the pixel design. Results of the current and the online study therefore conflict with studies that consider human eyes to be the strongest stimulus to reflexively direct the visual attention of an interaction partner due to their biological and social relevance (Tipper et al., 2008). The results also contradict studies that observed slower responses in direct comparisons of human and robotic eyes (Bonmassar et al., 2019).

Also, for the uncued trials, either of the anthropomorphic eye designs led to shorter reaction times compared to human eyes, and one of the anthropomorphic designs (cross) produced shorter saccadic latencies. Hereby results differ from the online study. As we did not change the stimuli it is hard to explain why the abstract anthropomorphic eyes supported attentional shifts in both, cued and uncued trials. This pattern of results is in contrast to the key mechanism of reflexive gaze cueing, which should always reveal shorter reaction times in cued trials compared to non-reflexive gaze cueing, but longer reaction times in invalid trials because of the higher effort to disengage attention (Ricciardelli et al., 2002; Friesen et al., 2004; Ristic and Kingstone, 2005). Thus, results still have to be further validated by future research to see whether abstract anthropomorphic eyes are the silver bullet in gaze cueing inducing only beneficial effects or whether the current results for the uncued trials do not represent a valid finding.

As was already discussed in more detail for the online study (Onnasch et al., 2022), the overall slower reactions to the human stimuli might have been due to a lack of saliency compared to the other stimuli because they were smaller (although the overall image size was the same) and less rich in contrast compared to the other cues. But this seems to be only half of the story, because if this was the exclusive driving force for the superior processing of the abstract anthropomorphic eyes then this should have also applied for the chunky, but purely symbolic arrows. Since this was not the case, it seems reasonable that the abstract anthropomorphic eyes combined best of both worlds. The anthropomorphic eye design triggered a social and therefore reflexive processing of the stimuli (Tomasello et al., 2007) while at the same time being easier to perceive than human eyes due the high contrast imagery.

To summarize results on reaction times and saccadic latencies, the findings are in favor of the abstract anthropomorphic eye designs as these eye gaze prototypes performed best in the cueing of attention.

The explorative analyses on the robot’s overall perception with the according stimulus prototypes favor an anthropomorphic eye design, too. Whereas no stimulus design discomforted participants, they attributed more competence to a robot with an anthropomorphic cross eye design. The perceived warmth of the robot was not significantly different but again descriptively higher for the anthropomorphic designs.

A clear limitation of this study is the small sample size. We aimed at 80 participants for a sufficient statistical power but had to halve the sample size because of an ongoing lockdown due to the COVID-19 pandemic. Some of the reported results just missed the conventional level of significance, which could have been a consequence of the small sample. Further studies are needed to replicate the current design with sufficient power. Another drawback with regard to transferability of results is that we used a highly controlled computer-based paradigm instead of engaging participants in an interaction with an actual robot. Our results therefore have to be interpreted as a first step to identify directional stimuli for robot design that support humans’ smooth attention reallocation in order to improve coordination in HRI. The current study did not represent a real human-robot collaboration. Naturalistic follow-up studies will have to validate the results in a real-world interaction and investigate whether the benefits of abstract anthropomorphic eyes persist and effectively ease the prediction of robot movements. In an actual working situation where people have to focus on other elements (such as assembly tasks), results may differ significantly which underlines the importance of more research. Another point to be considered in future studies is to parametrize and empirically explore the differences between the stimulus designs to better understand the underlying mechanisms leading to the observed effects. Lastly, since the stimulus condition was varied between subjects, no statement can be made about possible interindividual differences.

In sum, the current study supported previous findings of the online study, showing a clear tendency for superior processing of abstract anthropomorphic eyes. Both of the abstract eye gaze prototypes performed well in attentional cueing, yet, as the results were not consistent across all measures, neither of the prototypes stands out in particular. However, one of the designs received higher competence ratings, which makes it seem appropriate for the implementation in work-related settings. These insights on predictive visual stimuli are a first step to translate basic social mechanisms into useful design recommendations to ease the coordination in HRI.

## Data Availability

The raw data supporting the conclusions of this article are available at the OSF (osf.io/wue6d).
